# Crosstalk between Gut Microbiota and Cancer Immunotherapy: Present Investigations and Future Perspective

**DOI:** 10.34133/research.0600

**Published:** 2025-01-23

**Authors:** Yuhui Tang, Qiaoting Cai, Zhi Tian, Wenkuan Chen, Hailin Tang

**Affiliations:** ^1^ State Key Laboratory of Oncology in South China, Guangdong Provincial Clinical Research Center for Cancer, Sun Yat-sen University Cancer Center, Guangzhou, China.; ^2^ Taneja College of Pharmacy, University of South Florida, Tampa, FL, USA.

## Abstract

Gut microbiota is crucial for protecting the homeostasis of immune locally and systemically, and its dysbiosis is essentially correlated to tumorigenesis, cancer progression, and refractoriness to cancer treatments, including the novel immunotherapy. Increasing evidence unravel the intricate role of gut microbiota in reshaping tumor microenvironment and affecting the efficacy and toxicities of immunotherapy, which shed more light on the future applications of gut microbiota in efficacious biomarker and combination treatment of immunotherapy. To better grasp the underlying crosstalk between gut microbiota and immunotherapy, more experimental and clinical trials are indispensable for the customized gut microbiota-based treatments in cancer patients undergoing immunotherapy.

## Introduction

The occurrence of immunotherapy has induced a drastic transformation in treatment landscapes of cancers, from solely targeting cancer cells in tradition into unleashing and augmenting the antitumor immune system of patients, including immune checkpoint inhibitor (ICI), cancer vaccines, oncolytic virus, adoptive cell therapy (ACT), and immune cytokine therapy [1]. It has been proved that a host of cancer types has attained considerable benefits from immunotherapy, like melanoma, lung cancer, hematologic tumors, gastrointestinal cancer, and renal cell cancer [2]. Nevertheless, owing to the intricate tumor microenvironment (TME) of cancer patients, there have been various responses to immunotherapy and some cancer patients still obtain limited response to immunotherapy or have defective immune-related adverse events (irAEs). Therefore, it is pivotal to investigate more immunotherapy-related efficacious biomarkers and combination regimens to intensify curative effects of immunotherapy and diminish the emergence of irAEs.

Accumulating researches have unraveled that the commensal microbiota and its metabolites exert an intricate effect to tumorigenesis and cancer progression as well as anticancer therapies, especially immunotherapy, in a shift between pro-inflammation and anti-inflammation situation [3]. Gut microbiota not only serves as a solid indicator of cancer development but also essentially generates the efficacy and/or toxicity of immunotherapy by reshaping TME in patients with cancer. Hence, the dual role of gut microbiota profiling in cancer substantially contributes to the enhanced or detrimental antitumor immunity between responders and nonresponders to immunotherapy. Of note, it might be a novel biomarker to discriminate the responders by identifying the dominance of “beneficial” bacteria and a therapeutic strategy to reinforce the efficacy of immunotherapy by modulating gut microbiota to a favorable population.

## Character of Gut Microbiota in Cancer Immunotherapy

### Gut microbiota manipulates the efficacy of immunotherapy

Currently, increasing evidence have signified the engagement of gut microbiota in manipulating the immunotherapeutic efficacy through various mechanisms. For instance, Liu et al. [4] unveiled that *Eubacterium rectale* was considerably promoted in the melanoma patients effectively responding to anti-programmed cell death protein-1 (PD-1) immunotherapy, and that its elevated enrichment was significantly correlated to the favorable survival of melanoma patients. Furthermore, *E. rectale* activated natural killer (NK) cells by modulating the production of L-serine and the following Fos/FosL signaling pathway to potentiate immunotherapy [4]. Likewise, Matson et al. [5] disclosed that the particular gut microbiota profiling, such as *Bifidobacterium longum*, *Enterococcus faecium*, and *Collinsella aerofaciens*, was crucially enriched in the responders to PD-1 blockade therapy among metastatic melanoma patients, whose fecal material unexpectedly induced the effects of curbing tumor growth, enhancing T cell response, and maintaining anti-PD-1 therapy in germ-free mice with melanoma. Similarly, Mai and colleagues [6] deciphered that *Faecalibacterium*, *Odoribacter*, *Eubacterium_eligens_group*, and *Anaerostipes* were more abundant in the responders to neoadjuvant immunotherapy in esophageal squamous cell carcinoma and functioned via the production of short-chain fatty acids. As to chimeric antigen receptor (CAR) T cell therapy, Stein-Thoeringer et al. [7] identified that *Bacteroides*, *Ruminococcus*, *Eubacterium*, and *Akkermansia* were most significant in determining CD19–CAR-T responsiveness in patients. Interestingly, in Giampazolias et al.’s study [8], vitamin D improved responses to ICI treatment in murine model or human samples by altering microbiome composition in favor of *Bacteroides fragilis*. The results above hinted that the specific gut microbiota profiling indeed turned to be a novel predictor of responders to immunotherapy in clinical practice (Figure). However, there is no consensus among the specific “beneficial” or “harmful” bacterium in predicting efficacy of immunotherapy across the studies, which requires investigations.

**Figure.**
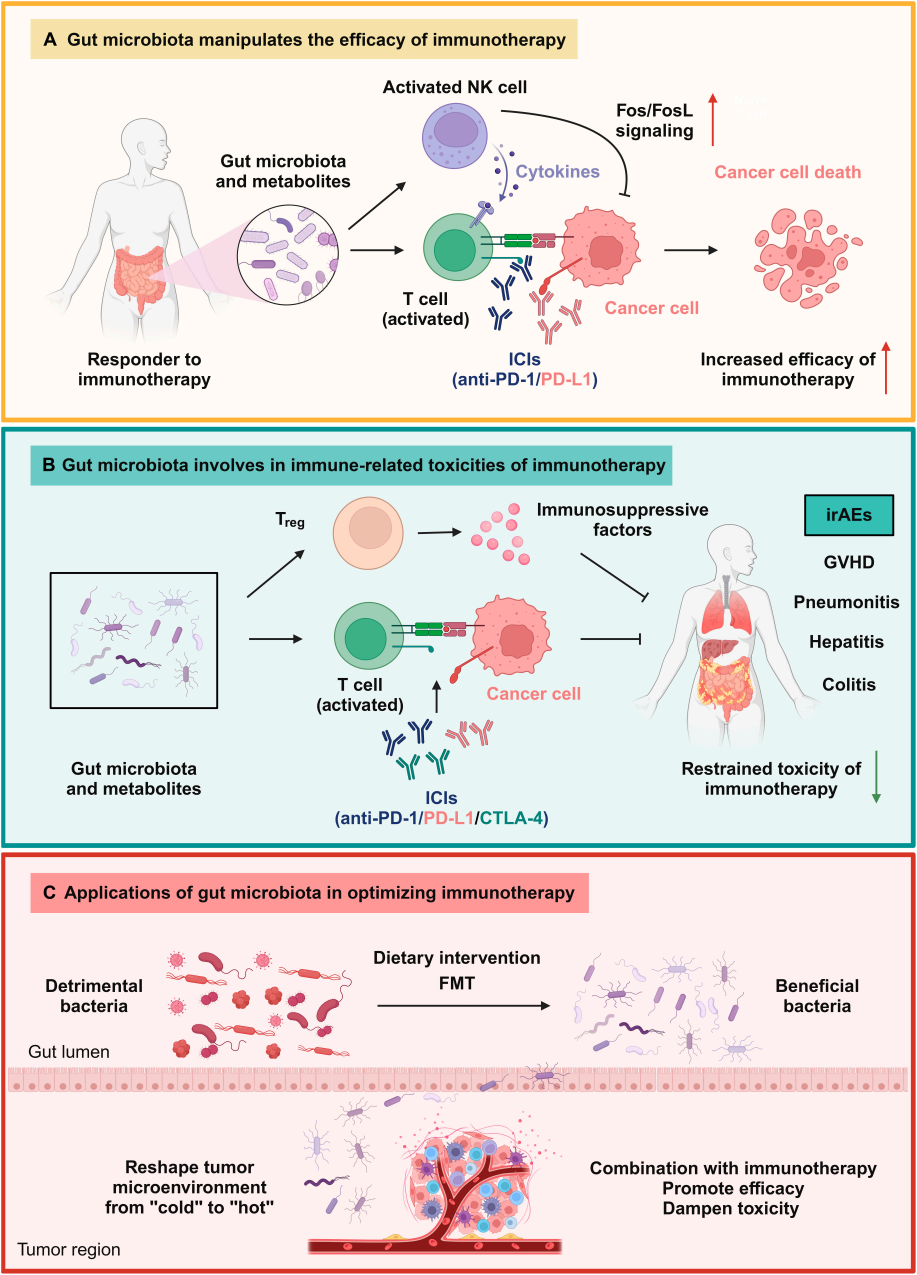
The underlying mechanisms of crosstalk between gut microbiota and immunotherapy. (A) Specific gut microbiota profiling and metabolites are enriched in the responders to immunotherapy among cancer patients, which enhances the efficacy of immunotherapy by activating NK cell through Fos/FosL signaling and thereby reinforcing the effects of activated T cell. (B) Beneficial gut microbiota diminishes the immune-related toxicities during immunotherapy through interaction with T_regs_. (C) Gut microbiota-based treatments including dietary intervention and FMT enable to change detrimental bacteria to beneficial bacteria, and reshape TME from “cold” to “hot”, which ultimately augment the efficacy and reduce the toxicity of immunotherapy. ICIs, immune checkpoint inhibitors; irAEs, immune-related adverse events; GVHD, graft-versus-host disease; FMT, fecal microbiota transplantation.

### Gut microbiota involves in immune-related toxicities of immunotherapy

It has been widely acknowledged that the function of immunotherapy is hindered in a host of cancer patients due to the occurrence of severe irAEs, including hepatitis, colitis, pneumonitis, and graft-versus-host disease (GVHD). The commensal microbiota was crucial in dampening hyperstimulation of the immune system and bringing about immune tolerance by boosting the accumulation of regulatory T cells (T_regs_) at mucosal barrier regions as well as inducing immunomodulating metabolites into circulation, which hinted that gut microbiota partly contributed to the immune-related toxicities [9]. Vétizou et al. [10] illustrated that the alteration of *Bacteroides* profiling induced by cytotoxic T lymphocyte-associated protein 4 (CTLA-4) blockade therapy was correlated to the severity of intestinal lesions in mice and patients, and that the efficacy and toxicity of anti-CTLA-4 therapy were uncoupled by gavage with *B. fragilis* in mice. Furthermore, Lo et al. [11] illuminated that gut microbiota composition was involved in the colitis induced by anti-CTLA-4 therapy, which was triggered by the unlimited activation of T cells and simultaneous attenuation of a subset of T_regs_ to interact with the Fc domain of the CTLA-4 antibodies via receptors. Another research depicted that the enhanced abundance of *Lactobacillaceae*, *Raoultella*, and *Akkermensia* was significantly linked to the decreased irAEs in advanced non-small cell lung cancer patients receiving anti-PD-1/PD-L1 therapies [12]. Collectively, the essential role of gut microbiota in the development of irAEs sheds more light on the reversal of the toxicities of immunotherapy in cancer patients (Figure).

### Applications of gut microbiota in optimizing immunotherapy in future

The traditional therapies, such as chemotherapies and radiotherapies, are generally executed in cancer patients before immunotherapy, which enable to generate the change of the commensal microbiota unfavorably. Accumulating researches have noted that it might be an effective combination therapy to manipulate the composition of gut microbiota to an optimized status of co-diversification, and a signature in human before receiving immunotherapy. Thus, several clinical trials to affect gut microbiota in patients undergoing immunotherapy have been carried out via dietary intervention and fecal microbiota transplantation (FMT) (Figure).

A previous study unveiled that the gut microbiota could be rapidly altered over a brief period during dietary changes, which indicated the feasibility of modulating gut microbiota by dietary intervention [13]. Moreover, Szczyrek et al. [14] reviewed that a diet enriched with sugar and fat in cancer patients was related to a worsened response to immunotherapy, and conversely, the consumption of dietary salt and fibers potentially elevated the immunotherapy efficacy. Mechanistically, the dietary fibers were fermented to produce some short-chain fatty acids like butyrate and propionate via specific gut microbiota, which served as the main metabolites to maintain immune homeostasis of intestines by interacting with the gut wall of effective responders to ICI therapy [15]. Additionally, high salt diet generated NK cell-mediated tumor immunity by restraining PD-1 expression and promoting interferon γ (IFNγ) and serum hippurate [16]. Particularly, salt effectively enhanced tumor immunity in combination with a suboptimal dose of anti-PD-1 therapy in melanoma-bearing mice [16]. Notably, 2 clinical trials (NCT04645680 and NCT04866810) are being performed to figure out the practicability of dietary intervention on affecting the efficacy and toxicity of ICI therapy in melanoma patients, which enlightens the application of customized diet with high fibers in patients undergoing immunotherapy. Nevertheless, the application of high salt diet in cancer patients undergoing immunotherapy calls for more clinical trials, especially when it is known that high salt intake adversely affects human health.

By assisting in recolonization of “healthy” bacteria derived from the donors to recipients by transplanting fecal material, FMT directly manipulated the gut microbiota and influenced the clinical outcomes of immunotherapy by maintaining microbial biodiversity [17]. In a phase I clinical trial involving 10 patients with metastatic melanoma who were refractory to anti-PD-1 therapy, 3 of them were proved to convert to responders to PD-1 blockade again after FMT, and none of them had severe irAEs [18]. Likewise, another first-in-human clinical trial recruiting 15 immunotherapy-refractory metastatic melanoma patients ciphered that 2 of those patients gained partial response, especially one who obtained complete response (CR) to PD-1 blockade therapy after FMT, despite the fact that all patients developed at least one irAE in a minimal degree [19]. Additionally, the personalized ecological topology score from gut microbiota in feces of cancer patients could represent intestinal dysbiosis and indicate the outcome of cancer immunotherapy [20]. Therefore, future investigations need to focus on the underlying mechanism of FMT functioning in combination immunotherapy.

## Concluding Remarks

By essentially impacting the functions of immune cells and reshaping the landscape of TME, synergistically combining with gut microbiota-based treatments and immunotherapy promisingly turns to be a novel therapeutic strategy in cancer patients. More importantly, more researches related to the application of gut microbiota in patients with different tumor types, lifestyle, comorbidities, and genetic inheritance during immunotherapy should be investigated in the future to better appreciate the crosstalk between gut microbiota and immunotherapy.
